# Design optimization of a contact-aided continuum robot for endobronchial interventions based on anatomical constraints

**DOI:** 10.1007/s11548-019-01972-8

**Published:** 2019-04-15

**Authors:** Laura Ros-Freixedes, Anzhu Gao, Ning Liu, Mali Shen, Guang-Zhong Yang

**Affiliations:** The Hamlyn Centre for Robotic Surgery, Imperial College London, London, SW7 2AZ UK

**Keywords:** Confined anatomy, Continuum robots, CCMs, Design optimization

## Abstract

**Purpose:**

A laser-profiled continuum robot (CR) with a series of interlocking joints has been developed in our center to reach deeper areas of the airways. However, it deflects with constant curvature, which thus increases the difficulty of entering specific bronchi without relying on the tissue reaction forces. This paper aims to propose an optimization framework to find the best design parameters for nonconstant curvature CRs to reach distal targets while attempting to avoid the collision with the surrounding tissue.

**Methods:**

First, the contact-aided compliant mechanisms (CCMs) are integrated with the continuum robot to achieve the nonconstant curvature. Second, forward kinematics considering CCMs is built. Third, inverse kinematics is implemented to steer the robot tip toward the desired targets within the confined anatomy. Finally, an optimization framework is proposed to find the best robot design to reach the target with the least collision to the bronchi walls.

**Results:**

Experiments are carried out to verify the feasibility of CCMs to enable the nonconstant curvature deflection, and simulations demonstrate a lower cost function value to reach a target for the nonconstant curvature optimized design with respect to the standard constant curvature robot (0.11 vs. 2.66). In addition, the higher capacity of the optimized design to complete the task is validated by interventional experiments using fluoroscopy.

**Conclusion:**

Results demonstrate the effectiveness of the proposed framework to find an optimized CR with nonconstant curvature to perform safer interventions to reach distal targets.

**Electronic supplementary material:**

The online version of this article (10.1007/s11548-019-01972-8) contains supplementary material, which is available to authorized users.

## Introduction

Interventional bronchoscopy is a rapidly maturing field which emphasizes advanced diagnostic and therapeutic bronchoscopy for the evaluation and management of life-threatening illnesses such as lung cancer [1]. During the intervention, a bronchoscope is inserted into the bronchi through the mouth or the nose to visualize its inside situation. It usually has an instrument channel, which is used by the practitioner to insert specialized tools, which usually consist of a flexible shaft and a distal steerable tip to perform different surgical procedures.

Due to the limitation of dimension and steerability, the commercial bronchoscope is usually only able to access the third and fourth generations of the respiratory tree, finally leading to unreachability to the deeper distal airways. It is also difficult to reach peripheral bronchioles by just using the traditional uncontrollable flexible tools going through the working channel, especially for the bronchi with acute bending angles. A wire-driven laser-profiled continuum robot with a central cavity has been proposed [2]. It consists of several segments with interlocked revolute joints. However, because all joints share the same bending range, its shape follows the constant curvature deflection, which increases the difficulty in entering specific airway bifurcations without relying on the passive reaction forces from the surrounding tissues. Thus, it is challenging to just use its own steerability to reach the distal airways. This inevitably increases the damage risk and compromises the patient safety.

There are some parameters of the robot design that can be optimized to form various robot shapes or tip trajectories, to finally accomplish a safer intervention in the confined anatomy. These include the joint number, the segment length and the maximum joint bending angles. The last one can be tuned by changing the cutout parameters to constrain the bending range for each joint to form CCMs [3]. By adjusting the bending limit for each joint individually, the joint self-contacts can occur at different times and locations while the robot deflects, thus allowing itself to achieve nonconstant deflections and a diverse range of shapes. The use of this joint self-contact-aided approach has already been explored and used for cardiac surgery in [4], where it is shown that the integration of CCMs has the potential of personalizing the continuum robot to perform specific tasks for endoluminal surgeries. A preliminary exploration of integrating CCMs to our developed laser-profiled continuum robot has been performed to accomplish the unidirectional bend for endobronchial intervention in [2]. Design optimization methods of continuum robots to perform specific medical tasks in confined space have previously been investigated. However, most existing work focus on concentric tube robots [5–9] and on a serial manipulator consisting in three links serially connected by revolute–prismatic joints [10], whose kinematic models differ noticeably from that of our cable-driven laser-profiled continuum robot.

In this study, we introduce the CCMs to our cable-driven laser-profiled continuum robot to form asymmetric deflections with the customized curvature, thereby adjusting the workspace and tip trajectory to adapt to the variable bronchi and confined anatomic space. This can help to reduce the collision with the anatomy and the associated risks of damage. A kinematics model considering self-contacts is formulated to describe its deflection accurately and efficiently, and then a model-based design optimization framework is built to find the best robot parameters to reach the specific target with the minimum collision in the confined anatomy. Finally, the optimization framework is tested on several distal airways to evaluate its performance in finding the best continuum robot configuration. The results are validated by implementing the intervention experiment using fluoroscopy. To our knowledge, this is the first time to integrate the laser-profiled continuum robot with CCMs to achieve the task-oriented design optimization toward the distal airways, thereby largely improving the safety during the intervention and enabling a potential disposable surgical instrument with a lower cost.

## Materials and methods

### Forward kinematics

**Fig. 1:**
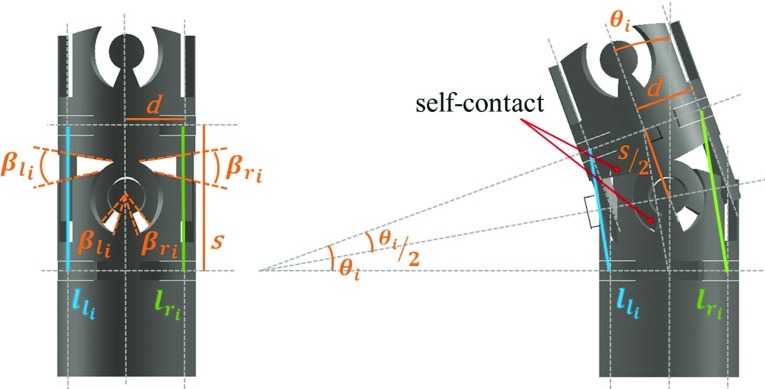
A schematic illustration of one segment of the cable-driven continuum robot with CCMs

The structure of the continuum robot is described in Fig. 1 by showing one robot segment with double CCMs. Robot segments can be defined from disk to disk. CCMs are implemented by adjusting the cutouts with $\beta _{\mathrm{l}}$ and $\beta _{\mathrm{r}}$ parameters to limit the joint maximal bidirectional bending angles. When one driven cable is pulled, but a segment reaches its bending limit, the deflection of this segment is fixed due to the self-contact of the joint mechanism, and it is assumed that the other segments will increase their bending angles continuously. To describe the 1-DOF deflection of the continuum robot, kinematics considering each segment’s constraints is built. The parameters of cutouts for each joint can be independently specified, which can further improve the robot’s deflection to adapt to diverse anatomical structures. The relationship between the lengths of the left and right cables—$l_{\mathrm{l}_i}$ and $l_{\mathrm{r}_i}$—and the joint bending angle $\theta _i$ of each segment is derived for a robot with *n* segments with CCMs:

1 $$\begin{aligned} l_{\mathrm{l}_i}= & {} 2\cdot (s/(2 \cdot \tan (\theta _i/2))-d) \cdot \sin (\theta _i/2) \end{aligned}$$

2 $$\begin{aligned} l_{\mathrm{r}_i}= & {} 2\cdot (s/(2 \cdot \tan (\theta _i/2))+d) \cdot \sin (\theta _i/2) \end{aligned}$$

3 $$\begin{aligned} \theta _i= & {} 2 \cdot \sin ^{-1}((l_{\mathrm{r}_i}-l_{\mathrm{l}_i})/(4 \cdot d)) \end{aligned}$$

where *s* is the length of the segment centerline, *d* is the distance from the center of the section of the robot to the center of actuation and $i \in [1,n]$ is the segment number, where *n* is the number of segments (see Fig. 1).

An algorithm is developed to find the deflection angle of each segment: given the robot number of segments *n*, the vector containing the bending limits for all the joints and the change of cable length $\Delta L_{\mathrm{l}}$ and $\Delta L_{\mathrm{r}}$ as the inputs, (i) calculate the total cable lengths $L_{\mathrm{l}}$ and $L_{\mathrm{r}}$ and obtain the cable lengths for each robot segment $i \in [1,n]$ with $l_{\mathrm{l}_i} = \frac{L_{\mathrm{l}}}{n}$ and $l_{\mathrm{r}_i} = \frac{L_{\mathrm{r}}}{n}$, which assume constant curvature, (ii) find each segment bending angle from its segment cable lengths by using Eq. (3) and (iii) check if any segment joint angle reaches its deflection limit. If not, stop and return the segment bending angles. If so, set the bending angle as the deflection limit and recalculate the associated segment cable lengths with Eqs. (1) and (2). Then, (iv) recalculate the cable lengths for the rest of the segments with equations $l_{\mathrm{l}_j}= \frac{L_{\mathrm{l}}-\sum _{k}l_{\mathrm{l}_k}}{n-{\text {numel}}(k)}$ and $l_{\mathrm{r}_j}= \frac{L_{\mathrm{r}}-\sum _{k}l_{\mathrm{r}_k}}{n-{\text {numel}}(k)}$, where *j* are the indexes of the subset of segments that do not reach their bending limit, *k* contains the indexes of the segments that reach its bending limit and ${\text {numel}}(k)$ is the total number of segments that reach its bending limit. (v) recalculate the deflection angles with Eq. (3) and repeat steps (ii)–(iv) until no further bending limits are reached. If all the joints reach its bending limit, there is no possible configuration.

**Fig. 2:**
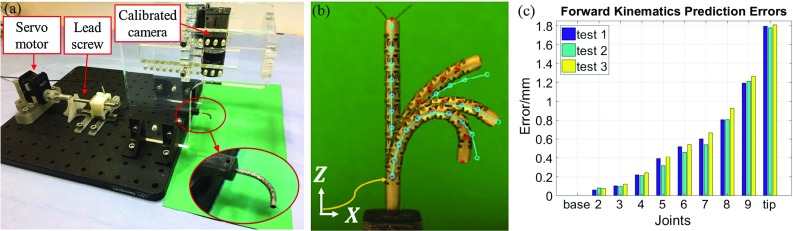
Kinematics modeling validation. **a** Experiment setup, **b** comparison of real (red) and predicted (blue) joint and tip positions for one deflection test, **c** joint and tip position average errors during deflection for three different deflection trials

The final shape of the robot is found by finding the position of the disks and the tip of the robot. The base reference frame of the robot is placed on the central position of the first disk $\mathbf {p}_{0},$ and it is considered to be at the origin of the world. The forward kinematics of the robot is formulated by concatenating transformation matrices to find the disk positions, $\mathbf {p}_{\mathrm{disk}}$, with respect to the base of the robot:

4 $$\begin{aligned}&\mathbf {p}_{\mathrm{disk}_{i}}= \varvec{T}_0^i \cdot \mathbf {p}_{0} ; i \in [1,n] \end{aligned}$$

5 $$\begin{aligned}&\mathbf {p}_{\mathrm{tip}} = \varvec{T}_0^n \cdot (\mathbf {{p}_{0}} + \mathbf {d}_{\mathrm{tip}}) \end{aligned}$$

6 $$\begin{aligned}&\varvec{T}_0^i = \varvec{T}_0^1 \cdot \varvec{T}_1^2 \cdot ... \cdot \varvec{T}_{i-1}^i \end{aligned}$$

where $i \in [1,n]$ is the disk index, $\mathbf {d}_{\mathrm{tip}}$ is the distance between the last disk and the tip of the robot, $\varvec{T}_0^i$ is the overall transformation matrix to obtain the position of the disk *i* and $\varvec{T}_{i-1}^i$ is the transformation matrix between the disk $i-1$ and *i*, which is shown in Eq. (7).

7 $$\begin{aligned} \varvec{T}_{i-1}^i= \begin{bmatrix} \cos \theta _i&\quad 0&\quad -\sin \theta _i&\quad -\sin \theta _{i} \cdot \dfrac{s}{2} \\ 0&\quad 1&\quad 0&\quad 0 \\ \sin \theta _i&\quad 0&\quad \cos \theta _i&\quad \cos \theta _i \cdot \dfrac{s}{2} +\dfrac{s}{2} \\ 0&\quad 0&\quad 0&\quad 1 \end{bmatrix} \end{aligned}$$

Forward kinematics considering the translation and the rotation along and around the longitudinal axis can also be implemented to represent the external rotation and the pushing/pulling of the robot through the bronchoscope.

### Model validation

A life-size prototype of the CR was manufactured according to Sect. 2.2 of [2]. The CR design parameters were chosen considering the human lung anatomy, the clinical requirements and the design constraints detailed in Sect. 2.1 of [2]. An unilateral bending experiment was designed to validate the forward kinematics model. The experimental setup used for our testing is shown in Fig. 2a. It consists of a Dynamixel MX-106 servo motor (Robotis, South Korea) connected to a lead screw to control the manipulator and an acrylic stand holding a calibrated high definition camera (Thorlabs, USA) above the manipulator to track its movement. Before the experiment, the motor was manually calibrated to a zero initial position, at which point, the manipulator was kept straight but any further motor rotation would lead to its bending. Then, the manipulator was bent to the right until it reached the bending limit and then retracted to the middle position. During the bending process, only the driven cable was pulled. The CR prototype had nine joints, and their maximum joint angles were set to $5^{\circ }$, $5^{\circ }$, $10^{\circ }$, $15^{\circ }$, $20^{\circ }$, $30^{\circ }$, $30^{\circ }$, $40^{\circ }$ and $40^{\circ }$, starting from the base. Deflection to the left was not allowed (maximum joint bending angles set to $0^{\circ }$).

Three tests were performed, in which the manipulator’s pose was captured for increasing variations of cable length. For each group, the cable was pulled up to 3.2 mm in 0.2 mm increments. The mechanism conveying error was considered. Around 17 images were captured for each test. The manipulator tip and joint centers were manually located in the images (Fig. 2b) and their positions were compared with the model for the same change of cable length. In Fig. 2c the average joint and tip errors between the real positions and the model positions are shown for each test. The largest errors occurred at the most distal joints of the robot in high bend configurations.

Several sources of error are identified. One of them is the manufacturing deficiency, which results in not very circular joints and inaccurate joint bending limits. Another source is the structure deformation that takes place when pulling the cable due to the material flexibility. In addition, the cable and the joint frictions are not modeled. The last source of error considered is the model assumptions. In the forward kinematics model, we assume that the joints that do not reach their bending angle limits follow the constant-curvature principle and have the same bending angle, which might not be applicable.

### Anatomical environment

The anatomical model is generated from CT images of the human lung. The lumen of the airway is segmented semi-automatically by using ITK-SNAP^1^ and is represented as a triangulated surface. For each bronchus, a centerline that connects its distal tip with the beginning of the airways is computed by using the vascular modeling toolkit^2^ (vmtk) and the bronchus section radius at each point of this centerline is obtained.

### Robot base definition and local frame

The reference frame of the continuum robot is located at the tip of the bronchoscope. The bronchoscope is assumed to follow the centerline to move toward the target point defined by a user in a distal area of the airways, as in [11]. The bronchoscope is assumed to be unable to move further when the radius of the bronchus section decreases to 3 mm, due to its tip diameter. The initial position of the tip of the bronchoscope is located at the point where the radius of the bronchus is 3.5 mm or 4 mm, and it is allowed to move following the centerline until the radius is decreased to 3 mm to adjust the tip position and orientation for a better robot deployment. The base of the robot is then positioned at the tip of the bronchoscope during the movement, and it is assumed to be on the centerline of the bronchus. The Frenet–Serret formulas are used to define the robot basis or local frame at a point $\mathbf {p_{0}}'$ on top of the 3D centerline by finding the tangent $\mathbf {T}$, normal $\mathbf {N}$ and binormal $\mathbf {B}$ vectors at this point. The origin of the robot is defined as the same as the origin of the anatomical world. The transformation matrix that expresses the required change of the robot base to place it at the point $\mathbf {p_{0}}'$ can be written as:

8 $$\begin{aligned} \varvec{T} = \begin{bmatrix} \mathbf {B}&\quad \mathbf {N}&\quad \mathbf {T}&\quad \mathbf {p_{0}}'\\ 0&\quad 0&\quad 0&\quad 1 \\ \end{bmatrix}. \end{aligned}$$

### Inverse kinematics

A discrete number of points of the bronchus centerline are defined as the desired CR tip trajectory for problem simplification, and the last point on this trajectory is set as the final target. Deriving a close form for the inverse kinematics is challenging when considering CCMs, as the constant curvature assumption cannot be applied. For that, we developed a semi-analytical method to calculate the shape of the robot when attempting to reach a particular target within its workspace. The 3-DOF deflection of the robot has been considered for that. Given a starting base position, we aimed to calculate the deflection of the robot, the external rotation and the base translation along the longitudinal direction that would allow the tip of the robot to reach a point.

Considering that the robot $x-y-z$ reference system is defined by the $\mathbf {B}$–$\mathbf {N}$–$\mathbf {T}$ vectors in the anatomical environment, the developed method consists of three steps. First, the robot is rotated around the direction defined by $\mathbf {T}$, so that its deflection plane, defined by the $\mathbf {B}$–$\mathbf {T}$ vectors, is aligned with the plane that contains the target. That way, the problem is reduced to 2D. Then, the deflection that aligns the *y*-coordinate of the tip position with the *y*-coordinate of the target is found. Finally, the difference between the *z*-coordinate of the robot tip position and the *z*-coordinate of the target is calculated and used for the robot translation along the direction defined by $\mathbf {T}$.

The required rotation angle is calculated by finding the angle between the normal vector of the current deflection plane and the normal vector of the desired deflection plane, as in Eqs. (9) and (10). The normal $\mathbf {n_{1}}$ of the current deflection plane is found by computing the cross-product of $\mathbf {B}$ and $\mathbf {T}$, while the normal of the desired deflection plane, $\mathbf {n_{2}}$, is found by computing the cross-product between the vector that connects the target point with the origin and $\mathbf {T}$.

9 $$\begin{aligned} |\alpha |= & {} \arctan {\frac{\left||\mathbf {n_{2}}\times \mathbf {n_{1}}\right||}{\mathbf {n_{2}} \cdot \mathbf {n_{1}}}} \end{aligned}$$

10 $$\begin{aligned} {\alpha }= & {} \text {sgn}(\left||\mathbf {n_{2}}\times \mathbf {n_{1}}\right||\cdot \mathbf {T})\cdot |\alpha | \end{aligned}$$

In order to deflect the robot to align its tip *y*-coordinate with the *y*-coordinate of the target, the maximum and minimum changes of cable length—$\Delta L_{\mathrm{min}}$ and $\Delta L_{\mathrm{max}}$—that the robot can hold given the CCMs constraints are computed. Then, given these cable length constraints, a constrained minimization problem can be formulated to find the deflection and robot shape that minimizes the distance between the tip *y*-coordinate $\mathbf {p}_{\mathrm{rot}\_\text {defl}\_\text {tip}_{y}}$ and the target *y*-coordinate $\mathbf {p}_{\mathrm{target}_{y}}$ in the local frame, which can be easily solved iteratively:

11 $$\begin{aligned} \begin{aligned}&\text {minimize}&\mathbf {p}_{\mathrm{rot}\_\text {defl}\_\text {tip}_{y}}-\mathbf {p}_{\mathrm{target}_{y}}\\&{\text {subject to}}&\Delta L \in [\Delta L_{\mathrm{min}},\Delta L_{\mathrm{max}}] \end{aligned} \end{aligned}$$

Allowing the base of the robot to move along the centerline makes this method not applicable, as the translation stops being linear. Hence, an iterative method is implemented to quickly find the required external rotation $\alpha$, deflection input $\Delta L$ and translation along the centerline $d_{\mathrm{centerline}}$ for the robot to reach a particular target at this stage of the trajectory. Although previous work had suggested the use of the gradient-based optimization solver *fmincon* from MATLAB^®^ to solve the inverse kinematics [12], this function was found to get stuck in local minima. Instead, the MATLAB^®^ global search method *GlobalSearch* was found to provide good results. Given a trajectory of *m* steps, we also aim to minimize the robot collision cost function at each step, $c_{j}(\mathbf {q})$, with $j \in [1,m]$. Redundant configurations need to be explored to find the deflection and translation that give the least collision. Thus, the inverse kinematics problem can be formulated as a nonlinear, constrained optimization problem:

12 $$\begin{aligned} \begin{aligned}&\mathbf {q}^{*} = \mathop {\hbox {argmin}}\limits _{q} c_{j}(\mathbf {q}) \\&\text {subject to: } ||{f(\mathbf {q})-\mathbf {p}_{\mathrm{target}_{j}}} ||= 0 \end{aligned} \end{aligned}$$

where $\mathbf {q}^{*}$ is the optimal set of parameters that defines the best motion plan to perform a step and reach the next step target $\mathbf {p}_{\mathrm{target}_{j}}$ and $f(\mathbf {q})$ is the function that uses the set of parameters $\mathbf {q}$ to find the robot tip position $\mathbf {p}_{\mathrm{tip}_{j}}$.

We calculate the robot collision with the anatomy at a particular step $c_{j}(\mathbf {q})$ with a delta ($\varDelta$) function that checks if the robot structure collides with the anatomy by checking whether a discrete number of points along the centerline of the robot are inside the eroded lumen of the airways, as in [9]. The center of the cable-guide disks and tip positions are used for this purpose. It is then defined as:

13 $$\begin{aligned} c_{j}(\mathbf {q}) = \sum _{i=1}^{n+1}\Delta ({\mathbf {p}}) \end{aligned}$$

where $\mathbf {p}$ represents the center of the disks and tip positions for that particular robot configuration defined by $\mathbf {q}$.

**Table 1:** Summary of the design optimization results

	Constant-curvature CR	Optimized CR
Fixed base		
Joint bending limits	$20^{\circ }$ for all the joints	$40^{\circ }$ for the first two joints. $5^{\circ }$ for the rest
Objective function	36.60	18.86
Moving base up to 5 mm		
Joint bending limits	$20^{\circ }$ for all the joints	$40^{\circ }$ for the first two joints. $5^{\circ }$ for the rest
Objective function	35.24	17.65
Moving base up to 7.5 mm		
Joint bending limits	$20^{\circ }$ for all the joints	$35^{\circ }$ for the first two joints. $5^{\circ }$ for the rest
Objective function	25.44	6.5
Moving base up to 10 mm		
Joint bending limits	$20^{\circ }$ for all the joints	$30^{\circ }$ for the first two joints. $5^{\circ }$ for the rest
Objective function	2.66	0.11

The objective function values have been scaled by dividing them by the maximum possible value and multiplied them by 100 for clarity

### Optimization

The design of the wire-driven continuum robot is defined as a set of parameters that are selected before its use and cannot be changed once it begins to perform a task. In this study, we have focused on the optimization of the joint bending angle limits of a single side of the robot, expressed with the parameter $\varvec{\beta }$. Thus, we considered that the robot would keep the original constant curvature deflection when deflecting to one direction (i.e., all joint bending angle limits equal to the same value), and we optimized the joint bending angle limits for the other deflection direction. In that way, we increased the number of shapes that the robot can achieve, which may be advantageous to reach multiple targets within the anatomy with the same robot design. We did not consider the optimization of other parameters such as the number of robot segments *n*, although this could be addressed by running our method several times for different number of segments. The method requires as input a specification of the environment geometry, the centerline of the desired bronchus, the location of the target point, the initial location of the base of the robot and the maximal distance that the robot base can move. For problem simplification, the user must also specify the number of joint sets in which the joint angle limits are divided. All the joints contained in the same joint set are assigned the same joint bending limit.

First, a list of all the parameter sets to be explored is created. The range of values for the joint bending angle limits will be kept between $5^{\circ }$ and $40^{\circ }$ to maintain the design stability. However, just a set of discrete values separated by intervals of $5^{\circ }$ is considered as possible joint bending angle limit values, due to the manufacturing errors produced by the laser profiling technique when cutting the tube.

For each parameter set to be explored, the inverse kinematics is computed and the associated objective function is calculated to compare its performance. We consider that for specific robot designs, the robot might not be able to complete the path and reach the target. We create an objective function that considers how much collision with the anatomy takes place while the robot tip follows the tip trajectory toward the target and penalizes the robot designs that cannot reach the target by calculating the percentage of steps unaccomplished by the robot. The total cost function becomes (notice that both terms are normalized):

14 $$\begin{aligned} C(\varvec{\beta },A,\text {path}) = \frac{\sum _{j=1}^{r}c_{j}(\mathbf {q})}{r\cdot {\text {numel}}(\mathbf {\mathbf {p}})} + \frac{|m-r|}{m} \end{aligned}$$

where $\varvec{\beta }$ contains the joint bending limits for each joint, *A* is the anatomical environment considered, $\text {path}$ is the tip trajectory considered, $\mathbf {q}$ is the robot configuration at a particular step, *m* is the total number of steps required to reach the target point, *r* is the total number of steps that the robot can perform along the path and ${\text {numel}}(\mathbf {\mathbf {p}})$ is the total number of points along the robot centerline that are used to check whether collision with the anatomy is taking place.

We aim to find the robot design that minimizes the objective function associated to its motion plan. For that, we consider the constraints associated with the robot design. Then, given the robot design parameters, the bronchial tree structure and the motion plan, we formulate the optimization problem as a constrained nonlinear optimization problem as follows:

15 $$\begin{aligned} \begin{array}{ll} {\text {minimize: }} &{}\quad C(\varvec{\beta },A,\text {path}) \\ \text {subject to}&{}\quad \beta _{i} \in [5^{\circ },40^{\circ }] \text {; } i\in [1,n] \\ &{}\quad \Delta L \in [\Delta L_{\mathrm{min}},\Delta L_{\mathrm{max}}]\\ \end{array} \end{aligned}$$

Once all the parameter sets have been explored, we define the parameter set with minimum objective function as the parameter set that defines the best robot design to reach a particular target.

## Use case example

### Simulations

We computationally demonstrate the effectiveness of our robot design optimization framework by considering the task of reaching different points of interest in the bronchial anatomy, while avoiding collision between the robot and the surrounding anatomical environment as much as possible. Here, we present an example in which we have optimized the robot joint angle limits to reach a target located in a distal area of a bronchus which cannot be reached by the bronchoscope due to the reduced diameter of the bronchus and its sharp bending angle. The anatomical model is generated from CT images of a human lung phantom, and two joint sets are considered for parameter set exploration.

The performance of the optimized design is compared with the performance of the state-of-the-art design that has constant curvature with joint bending angle limits equal to $20^{\circ }$. Initially, the robot base is placed at a point in which the bronchus radius is 4 mm and is assumed to be fixed. Then, the robot base is allowed to be moved up to 5 mm, 7.5 mm and 10 mm, at which point the radius is reduced to 3 mm. Optimized designs are obtained for each of the four cases to see the effect that the start location has on the optimization.

**Fig. 3:**
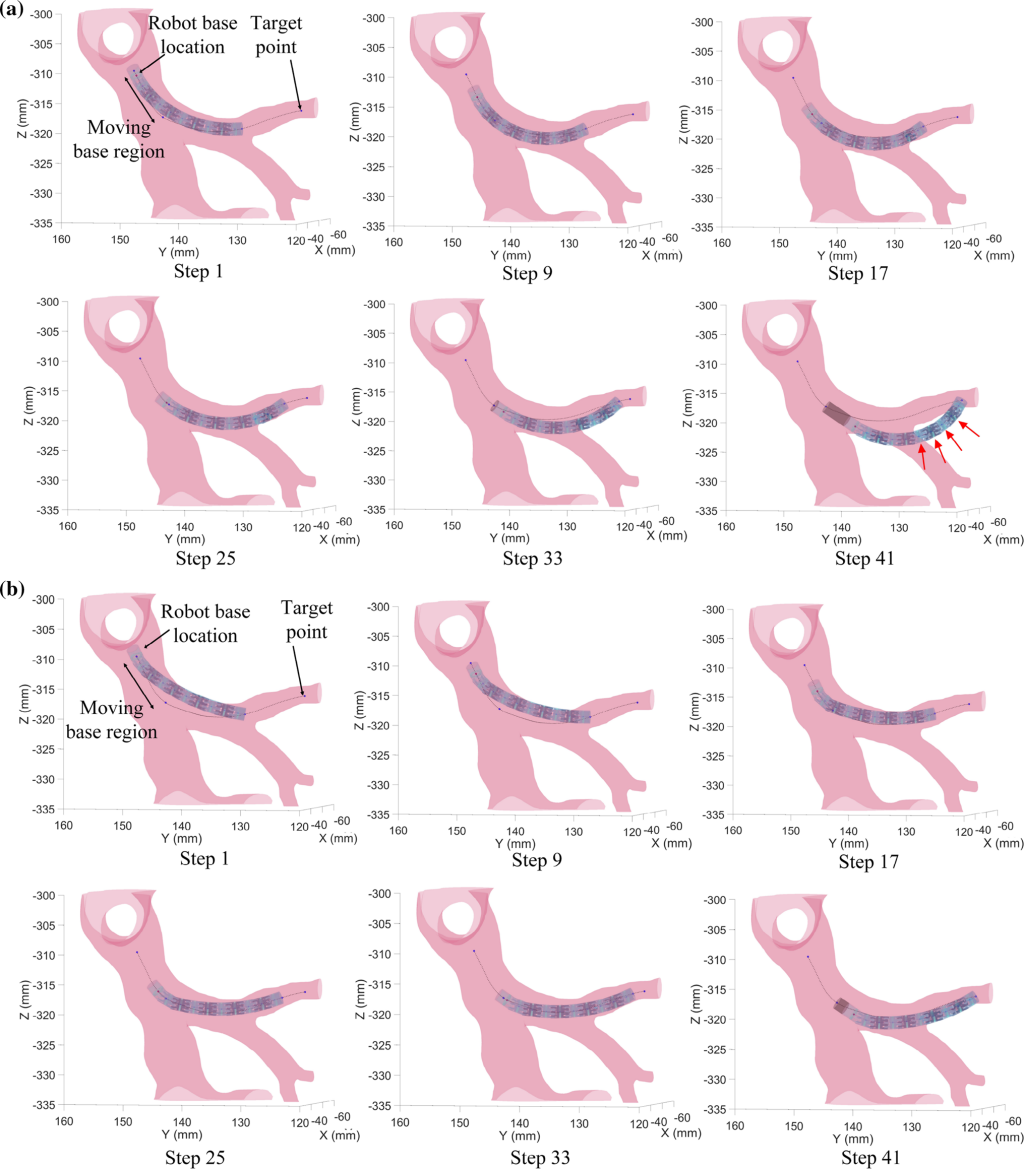
Simulation results for the CR with constant curvature (**a**) and the optimized CR (**b**) with moving base up to 10 mm

**Fig. 4:**
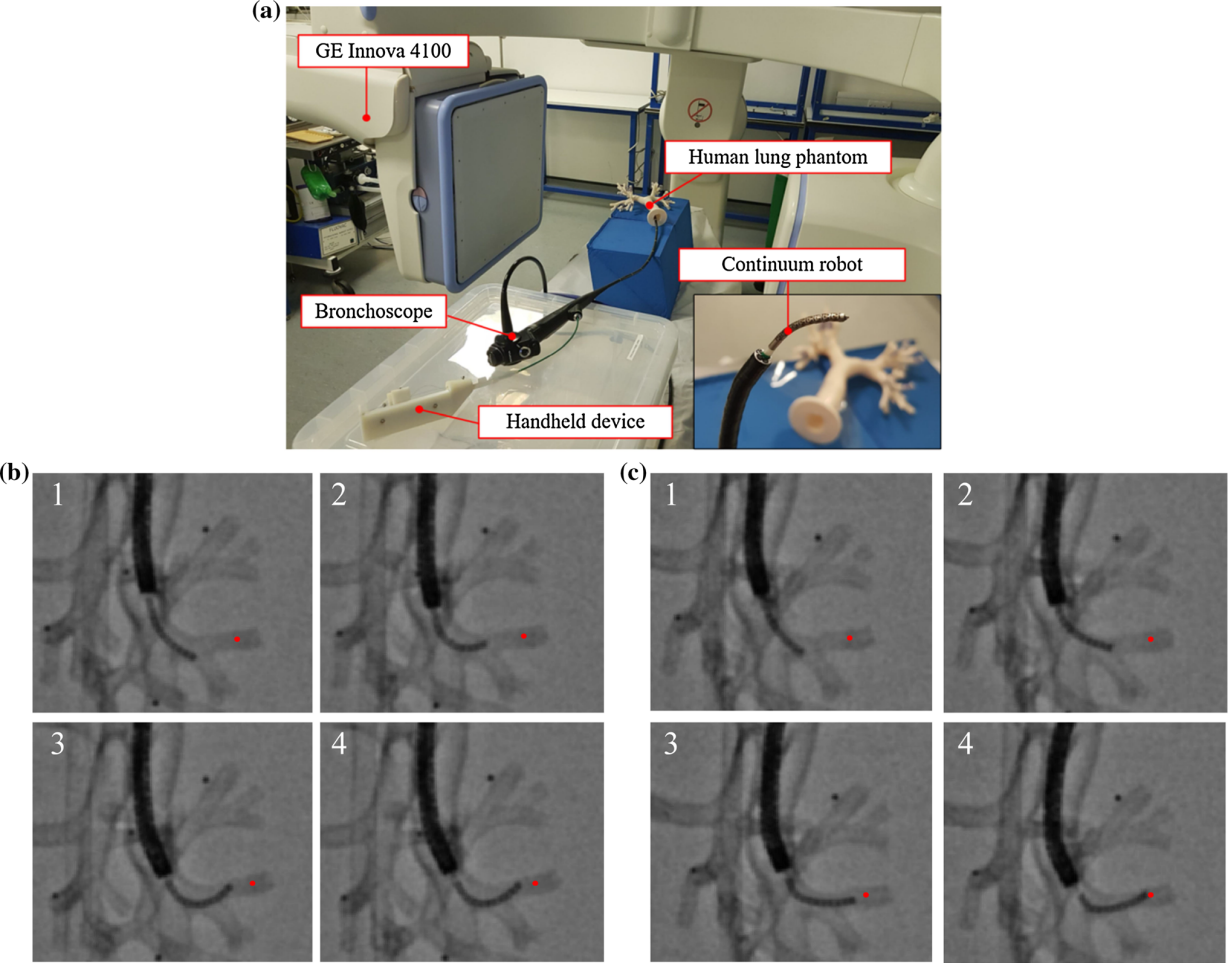
Optimization validation. **a** Experimental validation setup. **b** X-ray images of the intervention performed with the constant-curvature CR. **c** X-ray images of the intervention performed with the optimized CR. The desired target is marked in red

The resulting joint bending limits and objective function for all the cases are detailed in Table 1. For the case in which the base of the robot is fixed, the optimized design, which allows nonconstant curvature, can reach further than the constant-curvature design, which leads to a lower cost function with respect to the constant-curvature case. However, the target is not reached and a lot of collisions with the walls take place, specially at the last steps. When the robot base (i.e., bronchoscope tip) is translated up to 5 mm, 7.5 mm and 10 mm along the centerline, the minimum objective function value is obtained for the optimized robot whose base moves up to 10 mm. This makes sense as at this point, the orientation of the tip of the bronchoscope has changed considerably, which makes the robot translation direction be more suitable to reach the target. In this case, both the constant-curvature robot and the optimized one reach the target (see Fig. 3). However, the constant curvature one has more collision than the optimized design, which does not have the same joint bending limits for all the joints, offering nonconstant curvature deflection. Thus, we can conclude that for this case, the best result is obtained for a nonconstant curvature robot with joint angles limits being $30^{\circ }$ for the first two joints and $5^{\circ }$ for the rest, when the tip of the bronchoscope is placed 10 mm away from the initial position following the centerline.

Another simulation result for another bronchus is shown in the supplementary material to further validate the proposed method (see the attached video for the interventional procedures for different bronchi).

### Optimization validation

An interventional experiment was performed to validate the optimization results by inserting the robot prototype into a flexible silicone airway phantom through a commercial bronchoscope (Olympus BT-1T260) and controlling it manually from outside with a handheld device. The same soft silicon phantom with the exact dimension of a real human lung of the simulations was used as for the intervention, and the same airway and target were chosen to validate the simulations. The experimental setup used for our testing is shown in Fig. 4.

During the interventional procedure, the bronchoscope and the handheld device were controlled cooperatively by two operators to follow the specific trajectory. The two best CR of the simulations (i.e., the CR with the constant curvature and the one with the optimized curvature) were manufactured in life-size dimensions and used to implement the same interventional procedure as in the simulations, and meantime, the continuous X-ray images were recorded. Image sequences of the procedure are shown in Fig. 4b, c and the videos of the complete interventions are shown in the supplementary material. Notice that there is some deviation in the robot trajectory compared to the simulation due to the inaccurate manual control of the device.

A quantitative evaluation of the collision of the robots with the anatomical structures was performed. Collision with the wall was manually computed by counting the total number of robot disks and tips in contact with the anatomy in the images, and normalizing it by the total number of analyzed points, as in the simulations. The constant curvature robot scored 23% of collision and did not reach the target, while the optimized robot scored 12% and managed to reach the target. Therefore, the experiments showed the higher capacity of the optimized CR to reach the target, which demonstrated the benefit of using CCMs to achieve nonconstant curvature and the optimization of the robot design to improve the performance of the robot to reach a specific target.

## Discussion

The benefits of using optimized nonconstant curvature CRs to reach distal airways with fewer collisions have been shown. First, the concept of integrating CCMs with our continuum robot has been introduced and forward kinematics has been modeled to express the relationship between the change of cable lengths and robot shape. The errors of forward kinematics have been observed, which are mainly due to the constant curvature assumption, the cable interactions with the friction and the manufacturing deficiencies. An exact mechanical model considering the nonlinear properties could be developed in future to describe the deflection with higher accuracy.

Our simulations have been found sufficient to find an optimized robot design that would improve the reach of the target. In addition, the optimization framework developed has been demonstrated to be capable of optimizing the joint bending angles of the CR to reach specific targets with fewer collisions. The comparison of the performance of the constant-curvature CR and the optimized one has revealed its potential. However, we did not model the tissue reaction forces and we assumed the shaft of the robot to be rigid. It is admitted that the shaft of the continuum robot is flexible and inevitably bends when it collides with the anatomy. A tissue interaction model between the continuum robot and the soft tissue could be built, and other robot design parameters including the number of segments or the length of the interlocking segments could be considered in the optimization. This would improve the overall realism of the framework and help obtain more reliable results, which would further improve the safety of the patients.

## Conclusions

In this study, we have proposed a contact-aided laser-profiled continuum robot to successfully accomplish the customized constant/nonconstant curvature; also an optimization framework has been developed to derive the optimized structural parameters for a safer intervention toward the distal airways in the confined anatomy. The proposed forward kinematic model has described the deflection characteristics of the contact-aided continuum robot, which has been experimentally validated. A method to numerically solve the inverse kinematics has also been introduced. The model-based optimization framework has been successfully built to find the best joint limits for the CR to reach the distal airways with less collision. Several exemplary distal airways have been explored, and the results have been validated by the intervention experiment on a silicon human lung phantom using fluoroscopy. The optimized results have indicated the feasibility of a customized continuum robot toward the specific anatomy. To sum up, the proposed contact-aided laser-profiled continuum robot and the optimization framework are beneficial to help not only extend the reach of the conventional bronchoscope to access the distal airways, but also enable the customized continuum robot to adapt to the anatomical space with less collision.

In the future, the optimized contact-aided continuum robot will be combined with an imaging probe to accomplish the robotic tissue scanning for the diagnosis of early stage cancer. In order to introduce the respiratory movements into the optimization framework, 3D models of the anatomical structures at different points of the respiratory cycle will potentially be created by using 4D-CT scans.

## Electronic supplementary material

Below is the link to the electronic supplementary material.

Supplementary material 1 (pdf 29594 KB)

Supplementary material 2 (mp4 51362 KB)

## Footnotes

1 http://www.itksnap.org.

2 http://www.vmtk.org.
