# Supplementary material for: Design optimization of a contact-aided continuum robot for endobronchial interventions based on anatomical constraints
Source: Int J Comput Assist Radiol Surg. 2019 Apr 15;14(7):1137–46. doi: 10.1007/s11548-019-01972-8 (PMC6570708; doi:10.1007/s11548-019-01972-8)
Supplement: Supplementary file 1 — Supplementary material 1 (pdf 29594 KB) [file 11548_2019_1972_MOESM1_ESM.pdf]

Design Optimization of A Contact-Aided Continuum Robot for Endobronchial Interventions Based on Anatomical Constraints - Supplementary material

Supplementary Model Validation Figures

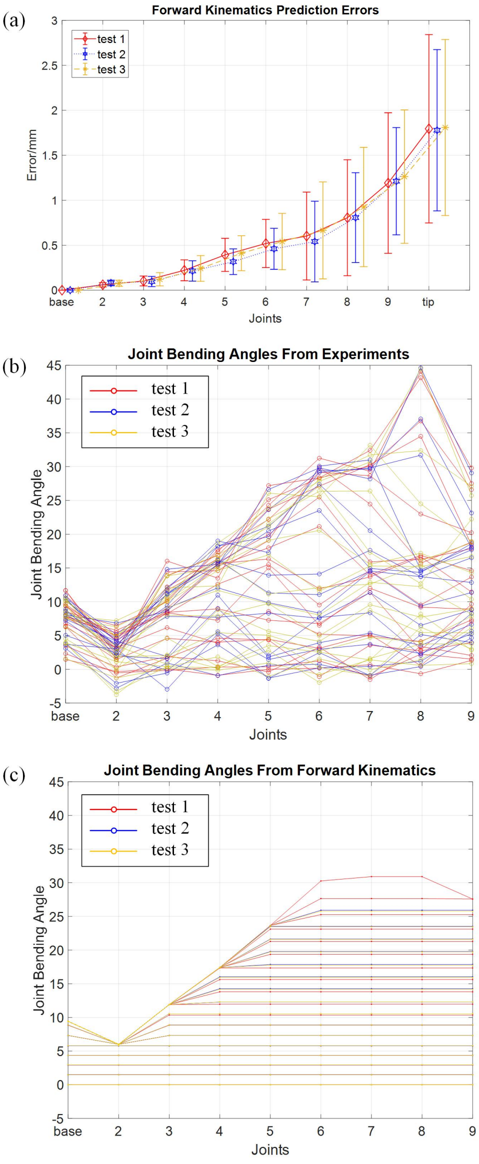

Fig. 1 Supplementary model validation figures. (a) Joint and tip position average errors during deflection for three different deflection trials with the standard deviation indicated. (b) Experimental joint bending angles during the bending tests. The different colours indicate the different tests. (c) Simulated joint bending angles during the bending tests.

Supplementary Use Case I Simulation Figures

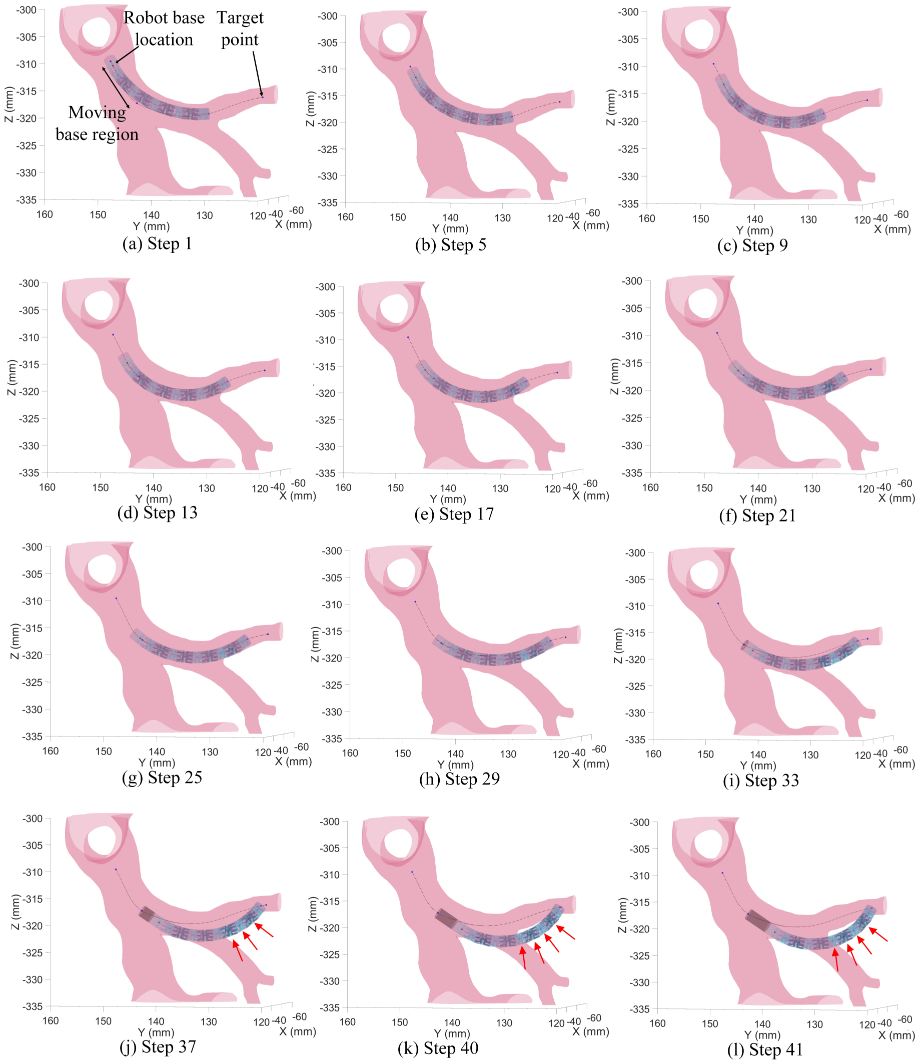

Fig. 2 Simulation results for the constant-curvature CR with optimized joint angle limits with moving base up to 10mm.

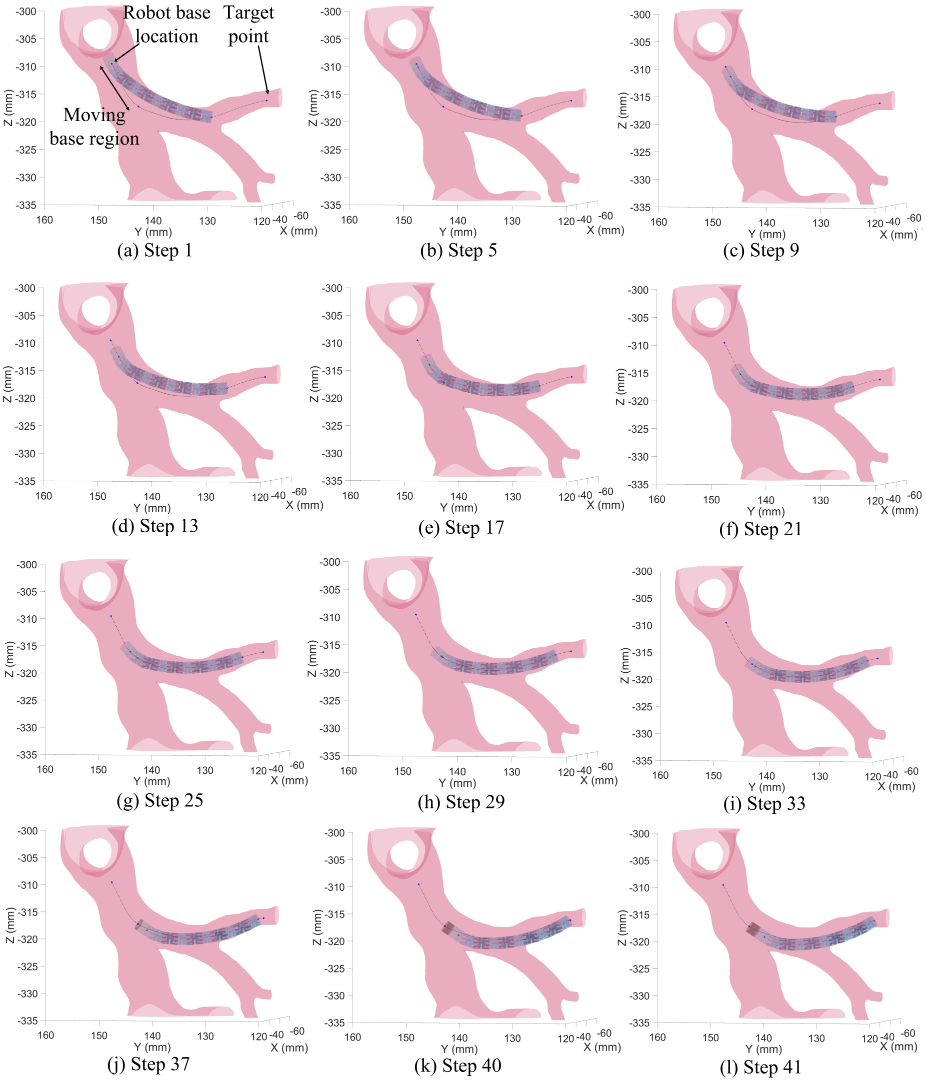

(a) Step 1 (b) Step 5 (c) Step 9
(d) Step 13 (e) Step 17 (f) Step 21
(g) Step 25 (h) Step 29 (i) Step 33
(j) Step 37 (k) Step 40 (l) Step 41

Fig. 3 Simulation results for the CR with optimized joint angle limits with moving base up to 10mm.

Use Case II

We have also optimized the joint angle limits of the continuum robot to reach a target located in a distal area of bronchus II, which cannot be reached by the bronchoscope due to the reduced diameter of the bronchus, and compared the performance of the optimized design with the state-of-the-art design that has constant curvature. Several base locations of the robot are tested; it is first assumed to be fixed and then translated up to 2.5 mm, 5 mm and 7.5 mm along the centerline as the bronchoscope is pushed further into the bronchus. The bronchoscope is not pushed more than 7.5 mm as the radius of the bronchus becomes too small for the bronchoscope to fit in. Optimized designs are obtained for each of the three cases mentioned. First, a table summarizing the joint bending limits and the objective function for each case is shown (Table 1). After that, sequences of images illustrating the motion plan of the constant-curvature robot and the optimized continuum robot for the cases with the lowest objective function are shown.

For the case in which the base of the robot is fixed, the optimized design, which allows non-constant curvature, can reach further than the constant-curvature design. However, the target cannot be reached and collision with the anatomy takes place, specially at the last steps. When considering that the base can be moved up to 2.5 mm, 5 mm and 7.5 mm, the result with minimum objective function value is obtained for the optimized design in which the robot base moves up to 7.5 mm (see Table 1). At this point, the orientation of the tip of the bronchoscope is changed considerably, which makes the robot translation direction be more suitable to reach the target (see Figs. 4 and 5). This allows the robot to reach the target for both the constant-curvature robot and the optimized one. However, for the constant curvature one, the collision is much higher than for the optimized design, which offers non-constant curvature deflection. Thus, we can conclude that for this case, the best result is obtained for a non-constant curvature robot with joint angles limits being 10° for the first two joints and 5° for the rest, when the orientation of the tip of the bronchoscope is adjusted by moving it up to 7.5 mm from the initial base position following the centerline.

		Constant-curvature CR	Optimized CR
Fixed base	Joint bending limits	20° for all the joints	25° for the first two joints. 5° for the rest.
	Objective function	35.15	25.20
Moving base up to 2.5mm	Joint bending limits	20° for all the joints	20° for the first two joints. 5° for the rest.
	Objective function	14.125	8.815
Moving base up to 5mm	Joint bending limits	20° for all the joints	15° for the first two joints. 5° for the rest.
	Objective function	13.85	9.16
Moving base up to 7.5mm	Joint bending limits	20° for all the joints	10° for the first two joints. 5° for the rest.
	Objective function	7.36	5.50

Table 1 Summary of the design optimization results for bronchus II. The objective function values have been scaled by dividing them by the maximum possible value and multiplied them by 100 for clarity.

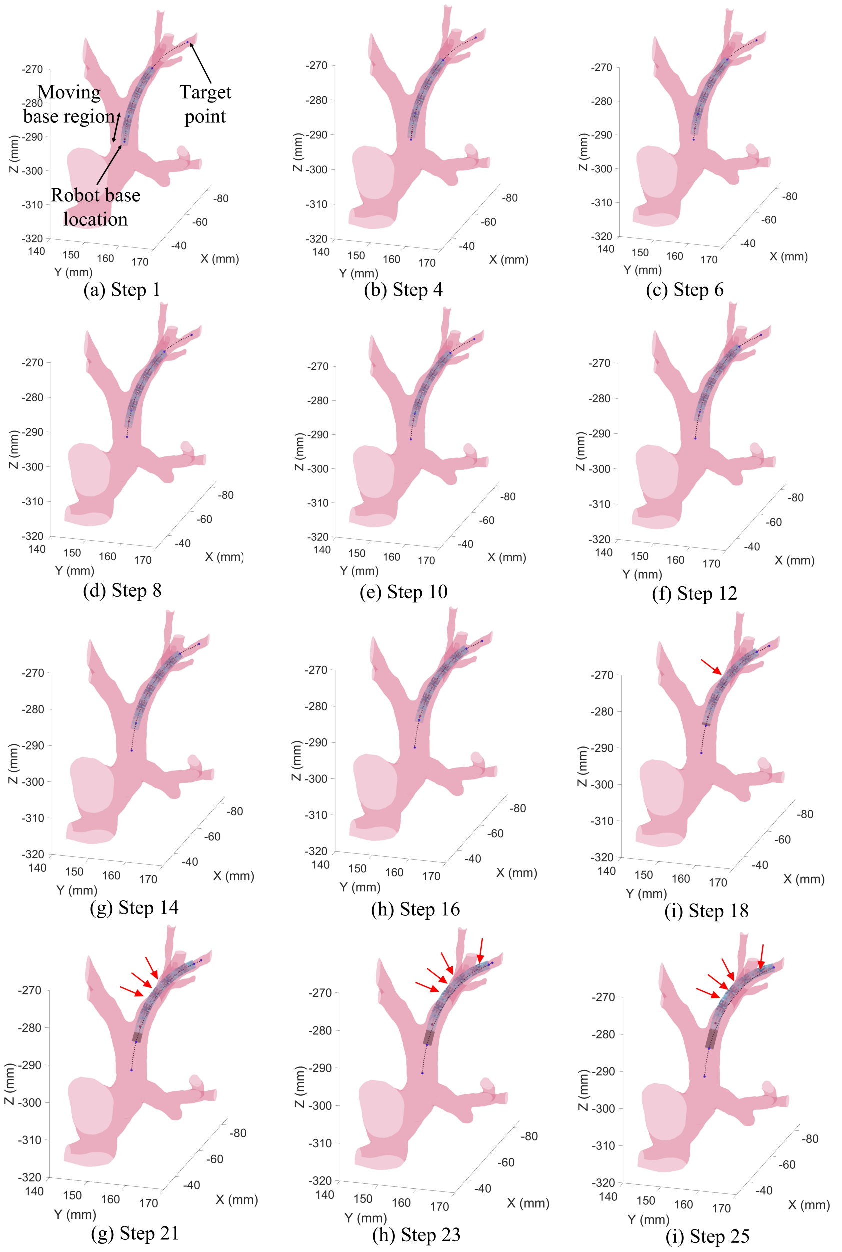

(a) Step 1 (b) Step 4 (c) Step 6

(d) Step 8 (e) Step 10 (f) Step 12

(g) Step 14 (h) Step 16 (i) Step 18

(g) Step 21 (h) Step 23 (i) Step 25

Fig. 4 Simulation results for the continuum robot with constant curvature with moving base up to 7.5 mm at bronchus II. Arrows indicate the points that are considered to be colliding with the anatomy.

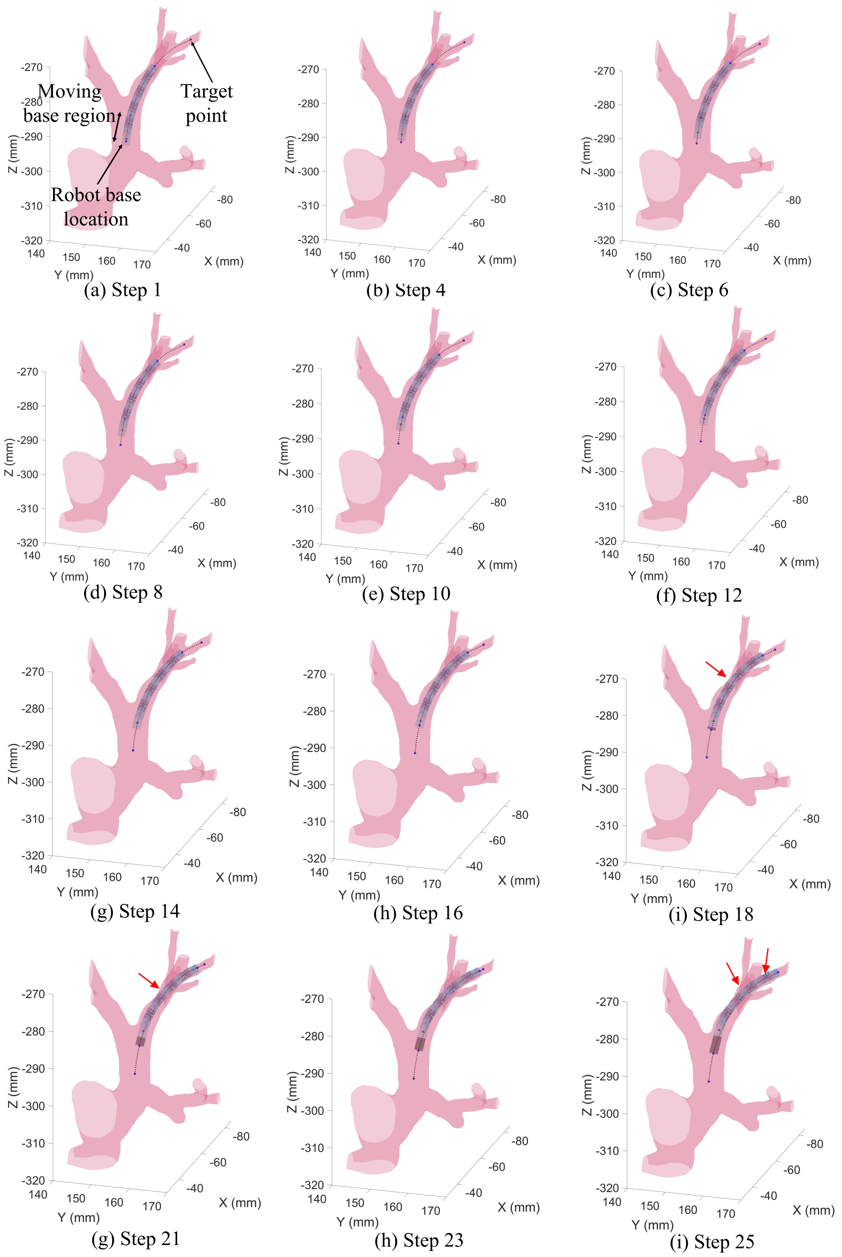

Fig. 5 Simulation results for the continuum robot with optimized joint angle limits with moving base up to 7.5 mm at bronchus II. Arrows indicate the points that are considered to be colliding with the anatomy.
